# Supplementary material for: The Progestin Medroxyprogesterone Acetate Affects HIV-1 Production in Human Lymphoid Tissue Explants in a Dose-Dependent and Glucocorticoid-like Fashion
Source: Viruses. 2021 Nov 18;13(11):2303. doi: 10.3390/v13112303 (PMC8621851; doi:10.3390/v13112303)
Supplement: Supplementary file 1 [file viruses-13-02303-s001.zip › viruses-1420054-supplementary.pdf]

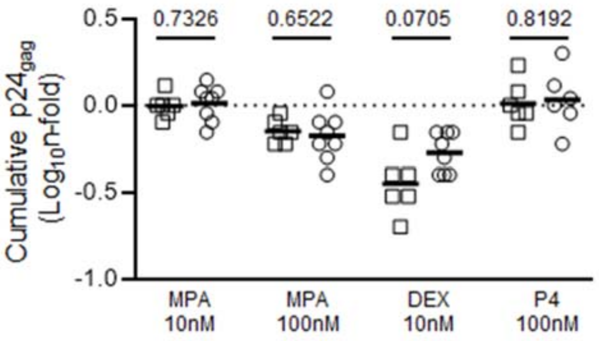

Supplementary Figure S1. HIV-1 cell tropism does not affect the changes in virus production associated with steroid hormone treatment of tonsillar tissue explants. Tissue explants were treated with medroxyprogesterone acetate (MPA), dexamethasone (DEX) and progesterone (P4) at the indicated concentrations overnight upon dissection. Culture supernatant was replaced with fresh medium containing MPA, DEX or P4 at 1 nM immediately before infection with HIV-1_{BaL} (CCR5-tropic) or HIV-1_{LAI} (CXCR4-tropic). Explants were cultured using the same medium composition for 12 days with a change of medium every 3 days as reported in Figure 1 (pre-infection). The chart represents values of n-fold change in cumulative virus production (day 6 to day 12 post-infection) in supernatant of treated explants normalized to donor-matched untreated explants infected with BaL (n=6, squares) or LAI (n=6-8, dots). Lines indicate mean values. A t test was used to compare the two groups.

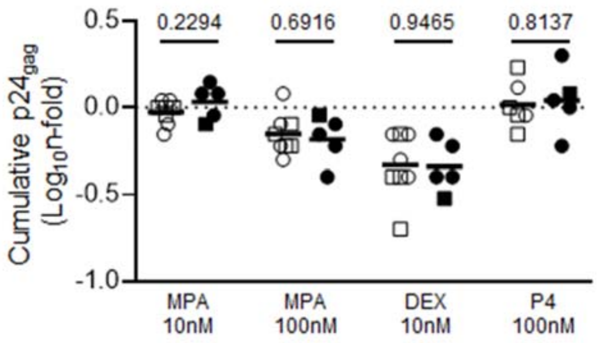

Supplementary Figure S2. Sex of tonsillar tissue donors does not affect the changes in HIV-1 production associated with steroid hormone treatment. Tissue explants were treated with medroxyprogesterone acetate (MPA), dexamethasone (DEX) and progesterone (P4) at the indicated concentrations overnight upon dissection. Culture supernatant was replaced with fresh medium containing MPA, DEX or P4 at 1 nM immediately before infection with HIV-1 and explants were cultured using the same medium composition for 12 days with a change of medium every 3 days as reported in Figure 1 (pre-infection). Results from HIV-1_{BaL} (squares) and HIV-1_{LAI} (dots) infection experiments were pooled for statistical analysis. The chart represents values of n-fold change in cumulative virus production (day 6 to day 12 post-infection) in supernatant of treated explants normalized to donor-matched untreated explants from female (n=6-8, white) and male (n=5, black) tissue donors. Lines indicate mean values. A t test was used to compare the two groups.

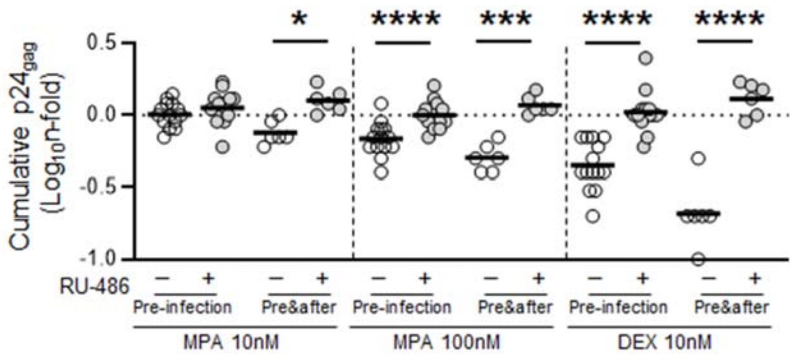

Supplementary Figure S3. Virus production in tonsillar tissue explants treated with steroid hormones. Tissue explants were treated with medroxyprogesterone acetate (MPA), dexamethasone (DEX) and progesterone (P4) at the indicated concentrations, in the presence or the absence of mifepristone (RU-486) at 1 µM, overnight upon dissection. Culture supernatant was replaced with fresh medium containing MPA, DEX or P4 at 1 nM (pre-infection, n=14), or containing the same amount of compound used for explant pre-treatment (pre&after, n=6), in the presence or the absence of RU-486 1 µM, immediately before infection with HIV-1. Explants were cultured using the same medium composition for 12 days with a change of medium every 3 days as reported in Figure 1 and Figure 2. Results from HIV-1_{BaL} and HIV-1_{LAI} infection experiments were pooled for statistical analysis.

The chart represents values of n-fold change in cumulative virus production (day 6 to day 12 post-infection) in supernatant of treated explants normalized to donor-matched untreated explants. Lines indicate mean values. Asterisks denote a statistically significant difference between each treatment group and the respective RU-486-treated control (two-way ANOVA with Sidak's multiple comparisons test, * $p<0.05$, ** $p<0.01$, *** $p<0.001$, **** $p<0.0001$).

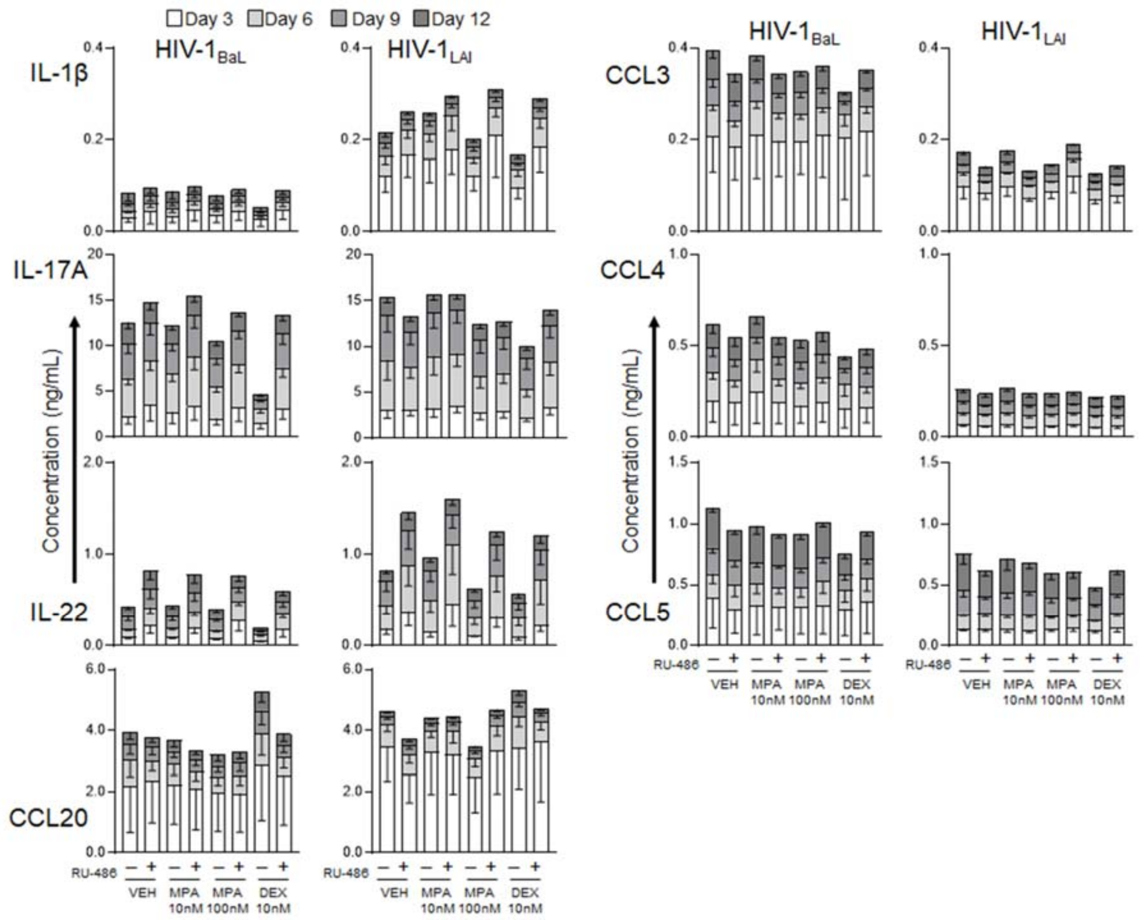

Supplementary Figure S4. Kinetics of cytokine production in HIV-infected tonsillar tissue explants treated with steroid hormones (pre-infection). Cytokine production was measured as the amount of cytokine released in culture supernatant of tonsillar tissue explants treated with ethanol 0.1% (VEH), MPA or DEX at the indicated concentrations, in the presence or the absence of RU-486 1 µM, prior to infection with HIV-1_{BaL} (n=5) and HIV-1_{LAI} (n=7). Culture supernatant was replaced with fresh medium containing MPA or DEX at 1 nM in the presence or the absence of RU-486 1 µM immediately before infection with HIV-1. Explants were cultured using the same medium composition for 12 days with a change of medium every 3 days as reported in Figure 1. Bars indicate mean values with s.e.m.
